# Usefulness of serum HBV RNA levels for predicting antiviral response to entecavir treatment in patients with chronic hepatitis B

**DOI:** 10.1007/s00535-025-02211-5

**Published:** 2025-01-22

**Authors:** Masanari Kosaka, Hatsue Fujino, Masataka Tsuge, Kenji Yamaoka, Yasutoshi Fujii, Shinsuke Uchikawa, Atsushi Ono, Eisuke Murakami, Tomokazu Kawaoka, Daiki Miki, Clair Nelson Hayes, Seiya Kashiyama, Sho Mokuda, Shinichi Yamazaki, Shiro Oka

**Affiliations:** 1 Department of Gastroenterology, Graduate School of Biomedical and Health Sciences, Hiroshima University, Hiroshima, Japan; 2 Liver Center, Hiroshima University Hospital, Hiroshima, Japan; 3 Section of Clinical Laboratory, Department of Clinical Practice and Support, Hiroshima University Hospital, Hiroshima, Japan; 4 Division of Laboratory Medicine, Hiroshima University Hospital, Hiroshima, Japan; 5 Department of Clinical Practice and Support, Hiroshima University Hospital, Hiroshima, Japan

**Keywords:** HBV RNA, HBsAg, HBeAg seroconversion, HBV DNA, Entecavir

## Abstract

**Background:**

Hepatitis B virus (HBV) RNA is an important serum biomarker of hepatic covalently closed circular DNA (cccDNA) transcriptional activity; however, its clinical characteristics remain unclear. This study evaluated the clinical utility of HBV RNA levels in patients with chronic hepatitis B (CHB).

**Methods:**

We studied 87 CHB patients with serum HBV DNA levels ≥ 5.0 log IU/mL who initiated entecavir (ETV) treatment between 2000 and 2018. Serum HBV RNA levels were measured at three-time points: before ETV treatment, at 12 weeks, and at 48 weeks after starting ETV treatment. Clinical markers associated with the antiviral effects of ETV treatment were analyzed.

**Results:**

Serum HBV RNA levels decreased in both HBeAg-positive and -negative patients during the observation period. In HBeAg-positive patients, multivariable analysis showed that lower HBV RNA levels at 48 weeks of ETV treatment were independently associated with HBeAg seroconversion. Additionally, lower baseline HBV RNA levels significantly predicted virologic response in those patients. In contrast, among HBeAg-negative patients, lower HBV core-related antigen (HBcrAg) levels and the FIB-4 index were independently associated with virologic response. In HBeAg-positive patients, those with higher baseline HBV RNA levels showed a more significant reduction in hepatitis B surface antigen levels.

**Conclusion:**

Serum HBV RNA levels predicted HBeAg seroconversion and early HBV DNA reduction in HBeAg-positive patients, while HBcrAg was significantly associated with virologic response in HBeAg-negative patients. These findings highlight the different predictive roles of HBV RNA and HBcrAg based on HBeAg status, which may provide individualized treatment strategies.

**Supplementary Information:**

The online version contains supplementary material available at 10.1007/s00535-025-02211-5.

## Introduction

Hepatitis B virus (HBV) infection is a serious global health concern. Although a universal vaccination program for preventing HBV infection has progressed globally, new HBV infections are estimated to occur in 1.5 million people, and 296 million people remain chronically infected [1]. Chronic HBV infection often leads to hepatitis, liver cirrhosis, and hepatocellular carcinoma (HCC), and the incidence of HCC in chronically infected individuals is 22.4-fold higher than that in uninfected individuals [2]. HBV genomes form covalently closed circular DNA (cccDNA) within hepatocytes, complicating complete viral eradication [3–6]. Thus, the purpose of antiviral treatment for patients with chronic hepatitis B (CHB) is to suppress viral replication and prevent liver disease progression using interferons and nucleoside/nucleotide analogs (NA) [7–9]. Current NA treatment effectively suppresses serum HBV DNA levels to undetectable levels. However, even if serum HBV DNA levels remain unmeasurable owing to NA treatment, the production of HBV-related proteins, such as hepatitis B surface antigen (HBsAg), continues and may be associated with hepatocarcinogenesis during NA treatment. Therefore, assessing intrahepatic HBV replication and identifying reliable viral markers are critical.

During the HBV life cycle, pregenome RNAs (pgRNAs) are derived from cccDNA and reverse-transcribed into plus-stranded genomic DNA in the core particle [10]. However, during NA treatment, reverse transcription of HBV RNA encapsulated in the core particle is suppressed by NA, and excessive accumulation of the HBV RNA particles might occur in hepatocytes. This accumulation induces HBV particle release into the blood, which includes HBV RNA [11–14].

We have previously reported an association between HBV RNA and drug resistance [11]. In another study, monitoring serum HBV DNA and RNA levels assisted in the prediction of HBV reactivation after the discontinuation of NA treatment [15]. Serum HBV pgRNA, a replication marker, correlates with cccDNA activity and decreases with NA treatment [16–18]. However, the association between changes in HBV serum markers, such as HBV pgRNA and HBV core-related antigen (HBcrAg), has not yet been clarified during NA treatment, and neither has its long-term antiviral effects. We measured serum HBV RNA levels before and during entecavir (ETV) treatment and evaluated the association between serum HBV RNA levels and antiviral effects in patients with CHB receiving ETV treatment.

## Methods

### Patients

This retrospective study included 158 patients who started ETV treatment between 2000 and 2018. We applied specific exclusion criteria. Patients co-infected with other viral infections, such as hepatitis C virus or human immunodeficiency virus, or those diagnosed with additional liver diseases, including autoimmune diseases, were excluded. Heavy drinkers with > 60 g/day ethanol intake were excluded to avoid the confounding effects of alcoholic liver disease [19]. We also excluded patients with a baseline HBV DNA level of less than 5.0 log IU/mL at the initiation of ETV treatment to focus on individuals with significant viral replication. Ultimately, 87 patients were eligible for analysis (Supplementary Fig. 1).

Blood samples were obtained, and serum HBV RNA was measured at baseline (before ETV treatment) and 12 and 48 weeks of ETV treatment. Biochemical and hematological tests were performed at our hospital. The remaining serum samples were stored at − 80 °C for further analysis.

All the patients provided written informed consent to participate in the study. The experimental protocol conformed to the ethical guidelines of the Declaration of Helsinki and was approved by the Ethics Committee of the Hiroshima University Hospital (Approved ID: E2022-0274).

### HBV RNA quantification

Circulating HBV RNA levels were measured using the Cobas HBV RNA real-time quantitative RT-PCR assay on a Cobas 6800 System (Roche Molecular Systems, CA, USA) [20, 21]. This assay has a lower limit of quantification (LLOQ) of 10 copies/mL and a linear range of 10–10^9^ copies/mL for armored RNA [21]. HBV RNA was considered undetectable if the result was < 0.5 log_10_ copies/mL.

### HBV-related marker quantification

HBsAg and HBeAg levels were measured using Abbott Alinity platforms (Abbott, Tokyo, Japan) as recommended by the manufacturer. The HBsAg assay range was 0.05–250 IU/mL. Further dilution was performed for samples with HBsAg levels exceeding 250 IU/mL to enable accurate measurement within the assay’s dynamic range. HBV DNA levels were measured using a real-time polymerase chain reaction (PCR) assay (COBAS® TaqMan HBV Test; Roche Diagnostics, Tokyo, Japan). The detectable range for HBV DNA quantitation was 1.3–8.2 log IU/mL. Serum HBcrAg levels were measured using iTACT-HBcrAg assay (Fujirebio Inc., Tokyo, Japan). The detection limit of the assay was 2.1 log U/mL. The upper limit of the measurement range was 7.0 log U/ml. For samples with HBcrAg levels exceeding 7.0 log U/mL, the samples were diluted with a sample dilution buffer and remeasured to ensure accurate quantification, following the manufacturer’s instructions.

### Primary outcomes

The primary outcomes were the association of HBV RNA levels and kinetics with virologic response, HBeAg seroconversion rate, and loss of HBsAg during ETV treatment. A “virologic response” was defined as HBV DNA < 1.3 log IU/mL. “HBeAg seroconversion” was defined as the loss of HBeAg (HBeAg-positive to HBeAg-negative status) and the appearance of HBeAb.

### Liver fibrosis indices

The fibrosis-4 index (FIB-4) was calculated according to a published formula (age [years] × aspartate aminotransferase (AST) [IU/L])/platelet count [10^9^/L] × alanine aminotransferase (ALT) [IU/L]^1/2^) [22].

### Statistical analysis

Categorical variables were presented as frequencies and continuous variables were presented as medians and interquartile ranges. The Spearman’s rank correlation coefficient was used to investigate the relationship between HBV RNA and other HBV markers, such as HBsAg, HBV DNA, and HBcrAg. Patients were classified into two groups according to cut-off values determined using receiver-operating characteristic (ROC) curves for the analysis of factors contributing to HBeAg seroconversion, and according to median values for the analysis of factors contributing to virologic response and HBsAg reduction. The predictive abilities of HBV RNA and HBcrAg levels for virologic response were compared using time-dependent receiver-operating characteristic (ROC) curves and the area under the curve (AUC). Univariable analysis using the log-rank test and multivariable analysis using the Cox proportional hazard model was used to identify the predictive factors contributing to HBeAg seroconversion and an early decrease in HBV DNA. We analyzed the data using continuous variables in multivariable Cox regression models. For all analyses, *P* < 0.05 was considered statistically significant. All statistical analyses were performed using EZR ver. 1.54 software (Saitama Medical Center, Jichi Medical University, Saitama, Japan) [23].

## Results

### Comparison of initial values between the HBeAg-positive and -negative groups

The baseline characteristics of the 87 patients are listed in Table 1. The median observation period was 8.3 years. No cases of virologic breakthrough or drug resistance were observed during the observation period. We divided patients into two groups according to their HBeAg status. Forty-eight HBeAg-positive and 39 HBeAg-negative patients with CHB were enrolled in this study. The HBeAg-positive group was significantly younger than those in the HBeAg-negative group (*P* = 0.002). Several HBV-related markers (HBsAg, HBV DNA, HBcrAg, and HBV RNA) in the HBeAg-positive group were significantly higher than those in the HBeAg-negative group (all *P* < 0.001). However, there was no significant difference in the FIB-4 index between the HBeAg-positive and -negative groups (*P* = 0.343).

**Table 1:** Baseline characteristics of the patients

Factors	Overall (*n* = 87)	HBeAg-positive (*n* = 48)	HBeAg-negative (*n* = 39)	*P* value**
Age (yr) *	49 (38–60)	43 (34–58)	56 (47–67)	0.002
Gender (male/female)	64 / 23	38 / 10	26 / 13	0.226
Platelet count (× 10^4^/µL) *	15.7 (13.0–20.4)	16.4 (13.5–21.2)	14.9 (11.5–19.1)	0.244
ALT (U/L) *	64 (38–174)	79 (49–212)	61 (31–132)	0.063
HBsAg (log IU/mL) *	3.6 (3.1–4.0)	3.8 (3.5–4.2)	3.1 (2.6–3.7)	< 0.001
HBeAg (positive/negative)	48/39			
HBV DNA (log IU/mL) *	7.2 (6.2–8.5)	7.6 (6.8–8.6)	6.5 (5.7–8.1)	< 0.001
HBcrAg (log U/mL)	6.4 (4.8–7.8)	7.6 (6.4–8.0)	4.3 (5.6–2.9)	< 0.001
HBV RNA (log copies/mL)	4.4 (2.9–6.1)	5.7 (4.4–6.8)	3.1 (3.8–2.3)	< 0.001
FIB-4 index*	2.2 (1.4–3.7)	1.9 (1.2–3.8)	2.0 (1.5–3.7)	0.343

*ALT* alanine aminotransferase, *HBeAg* hepatitis B e-antigen, *HBcrAg* hepatitis B core-related antigen, *HBsAg* hepatitis B surface antigen

^*^Median (interquartile range)

^**^Statistical analyses were performed by Fisher’s exact test or Mann–Whitney U test

### Correlations between HBV RNA levels and other HBV-related markers before ETV treatment

**Fig. 1:**
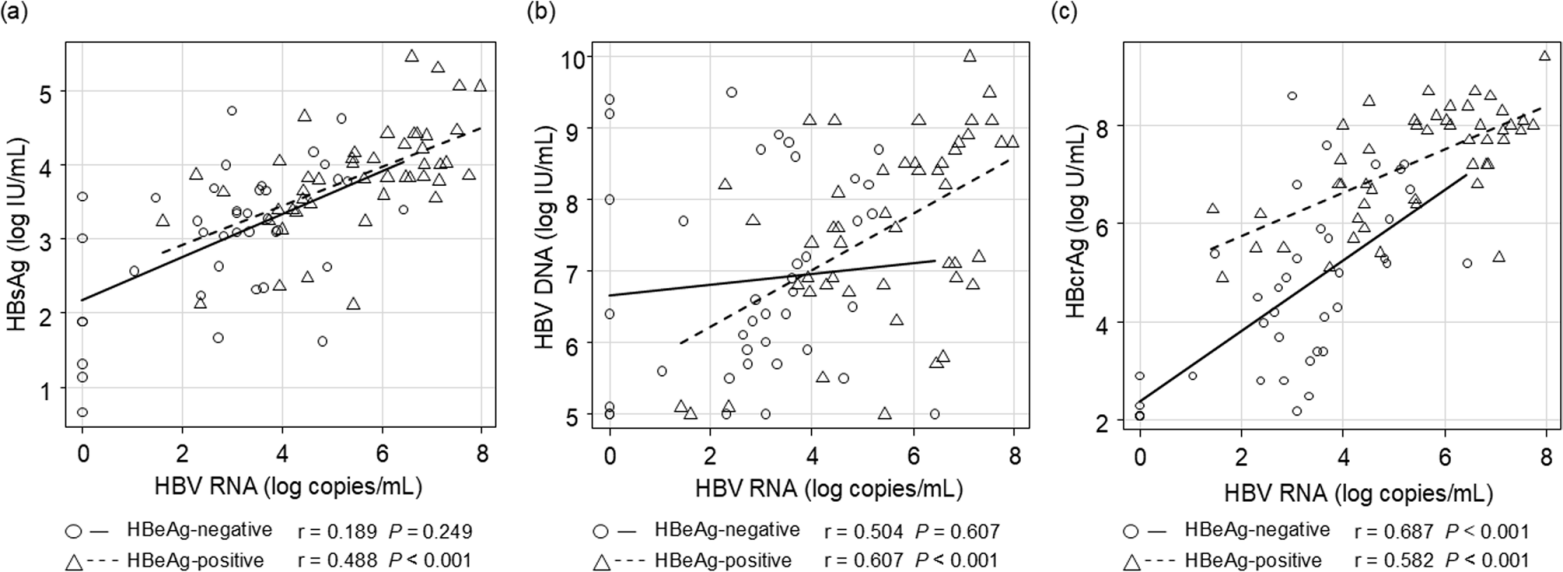
Correlation between hepatitis B virus (HBV) RNA levels and other HBV-related markers before entecavir (ETV) treatment. Correlations between HBV RNA levels, **a** hepatitis B surface antigen (HBsAg), **b** HBV DNA levels, and **c** hepatitis B core-related antigen (HBcrAg) levels in patients with chronic hepatitis B (CHB), indicated using scatter plots. The data points are distinguished based on hepatitis B e-antigen (HBeAg) status: circles represent HBeAg-positive patients, and triangles represent HBeAg-negative patients

The correlations between HBV RNA levels and other HBV-related markers at the initial visit were analyzed. HBV RNA significantly correlated with HBsAg (*r* = 0.488, *P* < 0.001), HBV DNA (*r* = 0.607, *P* < 0.001), and HBcrAg (*r* = 0.582, *P* < 0.001) in HBeAg-positive group. In the HBeAg-negative group, it only correlated with HBcrAg (*r* = 0.687, *P* < 0.001) (Fig. 1). We also investigated the correlations between HBV RNA, HBsAg, and HBV DNA at 12 and 48 weeks of ETV treatment. At week 12, HBV RNA correlated with HBV DNA in both HBeAg-positive and -negative groups, but not with HBsAg. At week 48, HBV RNA showed correlations with both HBsAg and HBV DNA in the HBeAg-positive group, while in the HBeAg-negative group, it only correlated with HBV DNA (Supplementary Fig. 2).

### Changes in serum HBV RNA level during ETV treatment

**Fig. 2:**
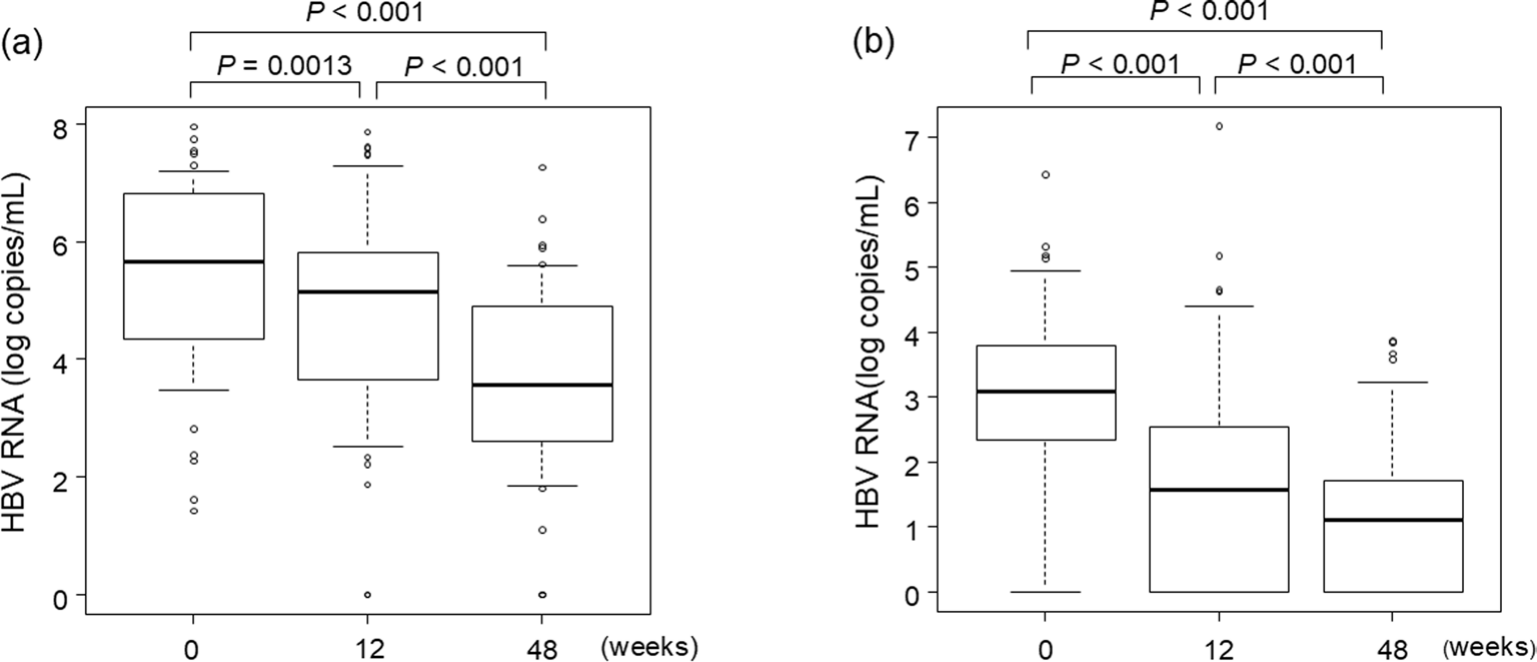
Changes in hepatitis B virus (HBV) RNA levels during entecavir (ETV) treatment. Changes in HBV RNA levels in **a** hepatitis B e-antigen (HBeAg) -positive and **b** HBeAg-negative patients at baseline and 12 and 48 weeks following ETV treatment initiation.

All enrolled patients initiated ETV treatment after their first visit. In the HBeAg-positive group, 12.5% and 66.7% of the patients achieved undetectable levels of HBV DNA after 12 and 48 weeks of ETV treatment, respectively. Median serum HBV RNA levels significantly decreased from 5.7 log copies/mL before ETV treatment to 5.1 log copies/mL and 3.6 log copies/mL after 12 and 48 weeks of ETV treatment, respectively (Fig. 2a). However, the HBV RNA reduction was more gradual than the HBV DNA reduction (Supplementary Fig. 3A).

In the HBeAg-negative group, 25.6% and 94.9% of the patients achieved undetectable levels of HBV DNA after 12 and 48 weeks of ETV treatment, respectively. Serum HBV RNA level also significantly decreased from 3.1 log copies/mL before ETV treatment to 1.6 log copies/mL and 1.1 log copies/mL after 12 and 48 weeks of ETV treatment, respectively (Fig. 2b). HBV RNA reduction was gradual compared to HBV DNA reduction (Supplementary Fig. 3B). HBV RNA reduction between HBeAg-negative and HBeAg-positive patients did not differ (*P* = 0.390).

### Association factors for HBeAg seroconversion in the HBeAg-positive group

Among the 48 HBeAg-positive patients, 30 (63%) patients achieved HBeAg seroconversion during the observation period. The median time from the start of ETV treatment to HBeAg seroconversion was 4 years. Statistical analyses were performed to identify the factors associated with HBeAg seroconversion. Univariable analysis revealed that age, ALT, HBcrAg, and HBV RNA levels before ETV treatment, and HBV RNA levels at 48 weeks of ETV treatment were associated with HBeAg seroconversion. The results of the Kaplan–Meier analysis, showing the cumulative HBeAg seroconversion rate stratified by baseline and on-treatment HBV RNA levels, along with the log-rank test, are presented in Supplementary Fig. 4. In multivariable analysis, only a lower serum HBV RNA level at 48 weeks of ETV treatment was identified as an independent factor associated with HBeAg seroconversion (*P* = 0.013) (Table 2).

**Table 2:** Factors associated with HBeAg seroconversion in the HBeAg-positive group

Variable	Univariable analysis	Multivariable analysis	
	*P* value*	HR (95% CI)	*P* value**
Age, ≥ 49/ < 49	0.004	1.003 (0.962–1.045)	0.901
Gender, male/female	0.922		
Platelet count (× 10^4^/µL), < 13.4 / ≥ 13.4	0.344		
ALT (U/L), < 56 / ≥ 56	< 0.001	1.000 (0.9984–1.001)	0.691
HBsAg (log IU/mL), < 3.8/ ≥ 3.8	0.115		
HBV DNA (log IU/mL), < 7.6/ ≥ 7.6	0.284		
HBcrAg (log U/mL), < 6.3/ ≥ 6.3	0.012	1.678 (0.748–3.765)	0.209
HBV RNA (log copies/mL), < 6.0/ ≥ 6.0	0.031	1.317 (0.743–2.335)	0.345
HBV RNA 12 weeks (log copies/mL), < 3.9/ ≥ 3.9	0.615		
HBV RNA 48 weeks (log copies/mL), < 3.7/ ≥ 3.7	0.001	0.650 (0.463–0.913)	0.013
FIB-4 index, < 1.1/ ≥ 1.1	0.371		

*ALT* alanine aminotransferase, *HBeAg* hepatitis B e-antigen, *HBcrAg* hepatitis B core-related antigen, *HBsAg* hepatitis B surface antigen

^*^Log-rank test

^**^Cox regression analysis

Patients who achieved HBeAg seroconversion had a more significant reduction in HBsAg levels at 1, 3, and 5 years after the start of ETV treatment than those who did not achieve HBeAg seroconversion (*P* = 0.048, 0.028, and < 0.001, respectively). However, the baseline HBsAg levels did not differ significantly (Supplementary Fig. 5).

### Association factors for ETV treatment-induced virologic responses

Forty-five HBeAg-positive (94%) and 39 HBeAg-negative (100%) patients achieved HBV DNA reduction to undetectable levels during ETV treatment (Supplementary Fig. 6). The median time to achieving HBV DNA levels below the detection limit was 266 days in HBeAg-positive patients and 112 days in HBeAg-negative patients. We examined the factors associated with earlier undetectable HBV DNA levels. In the HBeAg-positive group, low HBV RNA, HBsAg, and HBcrAg levels before ETV treatment were significantly associated with undetectable HBV DNA levels (*P* = 0.001, *P* = 0.004, and *P* = 0.006, respectively). Multivariable analysis identified lower HBV RNA levels significantly associated with virologic response (*P* = 0.001) (Table 3). Using time-dependent ROC curve analysis, the AUC for HBV RNA in predicting virologic response was higher than that for HBcrAg in HBeAg-positive patients (Supplementary Fig. 7A).

**Table 3:** Factors associated with virologic response in HBeAg-positive patients on ETV treatment

Variable (median)	Univariable analysis	Multivariable analysis	
	*P*-value*	HR (95% CI)	*P*-value**
Age, < 43/ ≥ 43	0.972		
Gender, male/female	0.079		
Platelet count (× 10^4^/µL), < 16.4 / ≥ 16.4	0.567		
ALT (U/L), < 79/ ≥ 79	0.567		
HBsAg (log IU/mL), < 3.8/ ≥ 3.8	0.004	0.909 (0.571–1.445)	0.686
HBV DNA (log IU/mL), < 7.6/ ≥ 7.6	0.163		
HBcrAg (log U/mL), < 7.6/ ≥ 7.6	0.006	0.736 (0.496–1.093)	0.130
HBV RNA (log copies/mL), < 5.7/ ≥ 5.7	0.001	0.553 (0.383–0.798)	0.001
FIB-4 index, < 1.1/ ≥ 1.1	0.838		

*ALT* alanine aminotransferase, *HBeAg* hepatitis B e-antigen, *HBcrAg* hepatitis B core-related antigen, *HBsAg* hepatitis B surface antigen

^*^Log-rank test

^**^Cox regression analysis

In the univariable analysis of the HBeAg-negative group, low HBV RNA, HBcrAg levels, and FIB-4 index before ETV treatment were significantly associated with early undetectable HBV DNA levels (*P* = 0.034, *P* = 0.001, and *P* = 0.015, respectively). Multivariable analysis revealed that lower HBcrAg levels and FIB-4 index were significantly associated with virologic response (*P* = 0.008 and *P* = 0.006, respectively) (Table 4). Time-dependent ROC curve analysis demonstrated that the AUC for HBcrAg was higher than that for HBV RNA in HBeAg-negative patients (Supplementary Fig. 7B).

**Table 4:** Factors associated with virologic response in HBeAg-negative patients on ETV treatment

Variable (median)	Univariable analysis	Multivariable analysis	
	*P*-value*	HR (95% CI)	*P*-value**
Age, < 56/ ≥ 56	0.060		
Gender, male/female	0.469		
Platelet count (× 10^4^/µL), < 14.9 / ≥ 14.9	0.142		
ALT (U/L), < 61/ ≥ 61	0.094		
HBsAg (log IU/mL), < 3.1/ ≥ 3.1	0.094		
HBV DNA (log IU/mL), < 7.0/ ≥ 7.0	0.079		
HBcrAg (log U/mL), < 4.3/ ≥ 4.3	0.001	0.673 (0.501–0.903)	0.008
HBV RNA (log copies/mL), < 3.1 / ≥ 3.1	0.034	0.805 (0.599–1.082)	0.151
FIB-4 index, < 2.2/ ≥ 2.2	0.015	0.736 (0.589–0.919)	0.006

*ALT* alanine aminotransferase, *HBeAg* hepatitis B e-antigen, *HBcrAg* hepatitis B core-related antigen, *HBsAg* hepatitis B surface antigen

^*^Log-rank test

^**^Cox regression analysis

### Association between HBV RNA levels and HBsAg reduction during ETV treatment

We investigated the relationship between serum HBV RNA levels before ETV treatment and the changes in HBsAg levels. In the HBeAg-positive group, HBsAg level was significantly decreased from 3.83 log IU/mL at the baseline to 3.56, 3.37, and 3.31 log IU/mL at 1, 3, and 5 years of ETV treatment, respectively. In the HBeAg-negative group, the HBsAg levels decreased significantly from 3.11 log IU/mL at baseline to 3.05 log IU/mL after 5 years of ETV treatment. However, they were not significantly different from the baseline at 1 and 3 years of ETV treatment (Supplementary Fig. 8).

**Fig. 3:**
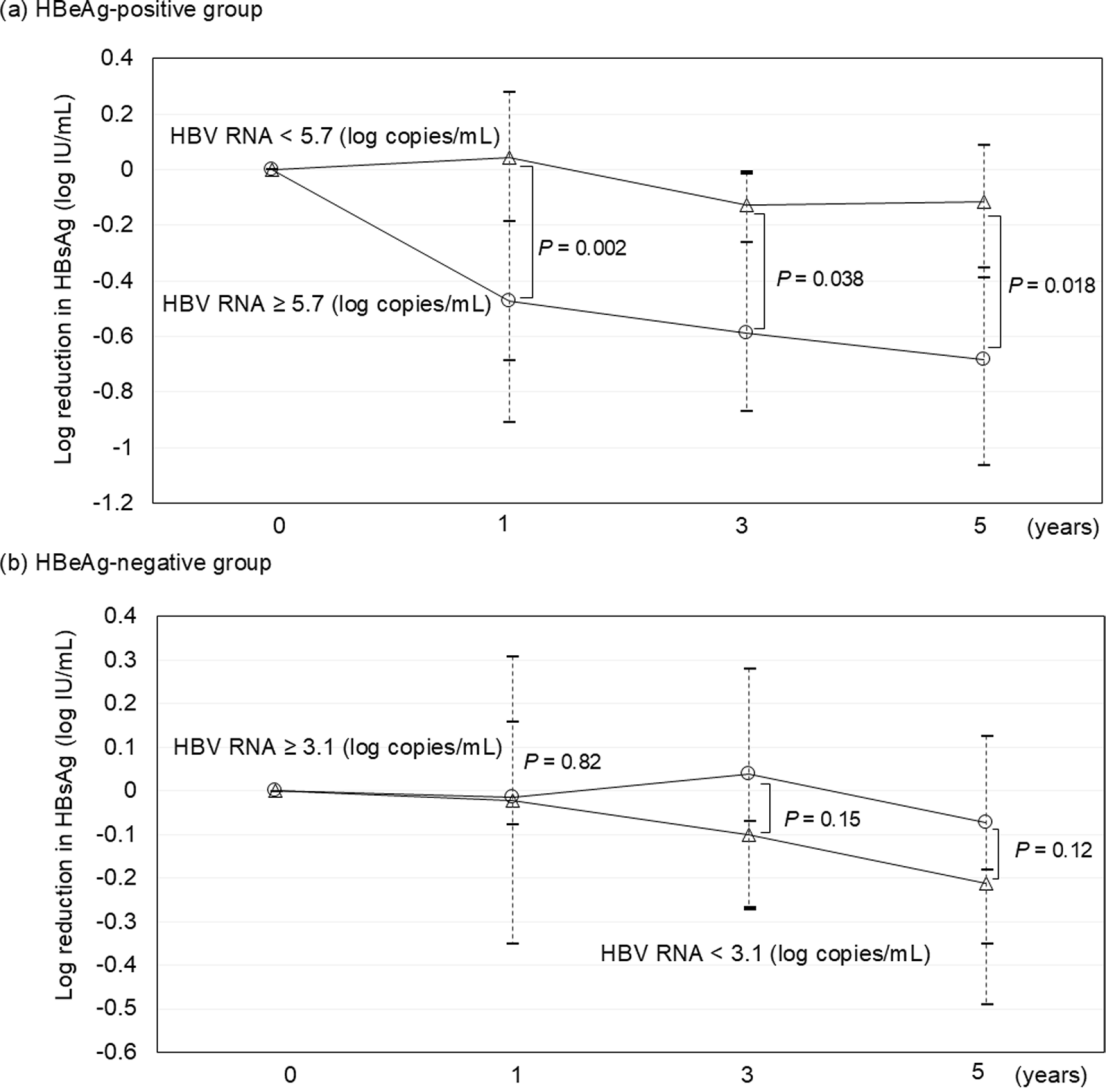
Changes of log reduction in hepatitis B surface antigen (HBsAg) by hepatitis B virus (HBV)-RNA levels. **a** In hepatitis B e-antigen (HBeAg)-positive patients, HBsAg decreased significantly in patients with high baseline HBV-RNA levels from 1 to 5 years following treatment initiation. **b** In HBeAg-negative patients, HBsAg reduction remained insignificant at baseline HBV-RNA level up until 5 years

Comparing the amount of HBsAg reduction in patients with high (≥ 3.1 log IU/mL) and low (< 3.1 log IU/mL) serum HBV RNA levels at the baseline in the HBeAg-negative group, there was no significant difference at 1, 3, and 5 years of ETV treatment (*P* = 0.82, *P* = 0.15, and *P* = 0.12, respectively) (Fig. 3b). Notably, in the HBe-positive group, patients with high serum HBV RNA levels (≥ 5.7 log IU/mL) at baseline had significantly more reductions in HBsAg levels than those with low baseline HBV RNA levels at 1, 3, and 5 years of ETV treatment (*P* = 0.002, *P* = 0.038, and *P* = 0.018, respectively) (Fig. 3a).

## Discussion

HBV RNA is an important serum biomarker reflecting transcriptional activity of hepatic cccDNA. However, its clinical characteristics remain unclear. To address this question, we evaluated changes in HBV RNA levels after initiating ETV treatment and examined the relationship between HBV RNA and the virologic response to ETV treatment. Before ETV treatment, HBV RNA significantly correlated with HBcrAg in both the HBeAg-positive and -negative groups. The correlation between HBV RNA, HBV DNA, and HBsAg during treatment differed between these two groups, suggesting potential distinctions in how HBV RNA reflects viral replication and antigen production.

During NA treatment, serum HBV RNA levels significantly and steadily decreased at 12 and 48 weeks in both patient groups, though more gradually than HBV DNA. This indicates that HBV RNA could be a reliable surrogate marker of cccDNA activity in patients with viral suppression. Both HBV RNA and HBcrAg reflect cccDNA activity, but they may capture different aspects of this activity. HBV RNA directly reflects the transcriptional activity of cccDNA, whereas HBcrAg reflects viral protein production. Thus, combining these markers could provide a more comprehensive assessment of cccDNA activity. Specifically, comparing their dynamics might enhance the prediction of treatment responses and inform biomarker selection based on patient status.

In HBeAg-positive patients, our study confirmed that low HBV RNA levels at 48 weeks of ETV treatment were a significant factor for early HBeAg seroconversion. Ji et al. demonstrated that the serum HBV RNA level 12 weeks after NA treatment was an independent prognostic factor for HBeAg seroconversion in patients with CHB with a high viral load after NAs treatment [24]. Luo et al. demonstrated that serum HBV RNA levels 24 weeks after NA treatment could predict the response to NA treatment in HBeAg-positive patients [25]. The discrepancy between these results may be attributable to several factors. First, differences in the patient populations and treatment protocols may have contributed to the varying results. In our study, HBV RNA at 48 weeks was a predictor of HBeAg seroconversion, with a median observation period of 8.3 years, which is a longer follow-up period than that in other studies, and the median time to HBeAg seroconversion was 4 years. Long-term observations may have strongly influenced these results. In addition, differences in the HBV RNA measurement methods and analytical approaches may have played a role. Differences in detection kits, sensitivities, and cutoffs used in different studies may lead to different results, even at the same time points. These factors may affect whether a particular time point is a significant predictor of seroconversion. Currently, no reliable markers can predict HBeAg seroconversion. Identifying such markers is crucial for predicting individual outcomes and establishing guidelines and initiation points for future therapeutic approaches.

As outlined in several guidelines [26–29], the treatment goals for HBV infection focus on reducing the morbidity and mortality associated with chronic HBV infection. The main goals include achieving sustained suppression of HBV DNA, a functional cure, and the disappearance of HBsAg. Our study highlights the potential of HBV RNA as a biomarker for predicting treatment response in patients with CHB receiving ETV treatment. Although HBV DNA, HBsAg, and HBcrAg are established markers, our findings indicate that HBV RNA levels provide additional, clinically relevant insights. For example, in HBeAg-positive patients, low HBV RNA levels at 48 weeks of ETV treatment were associated with higher HBeAg seroconversion rates. Furthermore, this study suggests different roles for HBV RNA and HBcrAg as predictors of virologic response. Lower baseline HBV RNA levels were linked to early HBV DNA suppression in HBeAg-positive patients, whereas lower baseline HBcrAg levels were significantly associated with virologic response in HBeAg-negative patients. Using time-dependent ROC curve analysis, the AUC for HBV RNA in predicting virologic response was higher than that for HBcrAg in HBeAg-positive patients, whereas HBcrAg demonstrated a higher AUC than HBV RNA in HBeAg-negative patients. These findings could be helpful for personalized treatment plans, identifying patients likely to respond favorably and aiding monitoring strategies.

We observed that HBsAg levels significantly decreased over time in HBeAg-positive patients, but only after 5 years in HBeAg-negative patients. Higher baseline HBV RNA levels in HBeAg-positive patients were associated with greater HBsAg reduction. Additionally, among the HBeAg-positive patients, there was no significant difference in baseline HBsAg levels between those who achieved HBeAg seroconversion and those who did not. However, HBsAg levels significantly decreased in those who achieved HBeAg seroconversion during ETV treatment. These findings suggest that HBV RNA and HBsAg levels may reflect different aspects of viral activity. First, lower baseline HBV RNA levels may indicate already suppressed viral replication, making these patients likely to achieve rapid viral suppression with NA treatment, subsequently leading to HBeAg seroconversion. In contrast, patients with high baseline HBV RNA levels might correspond to greater HBsAg reduction, possibly reflecting a more pronounced immune response. While our study does not establish a direct mechanistic relationship between baseline HBV RNA levels and HBsAg decline, the data suggest that baseline HBV RNA levels may provide useful prognostic information for predicting HBsAg reduction, specifically in HBeAg-positive patients. The significant association between higher baseline HBV RNA levels and greater HBsAg reduction observed only in HBeAg-positive patients, but not in HBeAg-negative patients, highlights the complexity of HBV infection and suggests that the relationship between HBV RNA and HBsAg may vary depending on HBeAg status. These findings emphasize the need for further research to clarify the mechanistic link between HBV RNA and HBsAg reduction.

As NA-mediated HBV polymerase inhibition blocks HBV replication by inhibiting the generation of reverse-transcribed rcDNA from pgRNA, it may not inhibit HBV pgRNA virion generation. The presence of pgRNA virions in serum reflects ongoing cccDNA transcriptional activity. Antiviral treatment with potent HBV polymerase inhibitors significantly reduced cccDNA levels in patients with CHB [30]. The decrease in intrahepatic cccDNA was primarily owing to the potent suppression of viral DNA synthesis, which effectively depleted the pool of mature cytoplasmic nucleocapsids available for conversion to cccDNA [31, 32]. NA achieves potent viral suppression but requires prolonged treatment in patients with CHB. In contrast, peg-interferon (PEG-IFN) sustains a virologic response after treatment ends, and notably, HBsAg disappears and is ultimately considered a “functional cure” [33, 34]. HBV RNA levels significantly decrease during PEG-IFN treatment in patients with HBeAg-negative CHB and correlate with HBsAg loss during long-term follow-up [35, 36]. Regarding HBV-RNA and HBsAg reduction during NA treatment, Liao et al. reported that HBV RNA levels significantly correlate with HBcrAg levels, but not HBsAg levels, during long-term NA treatment [37]. The differences in the relationship between HBV RNA and HBsAg reduction observed with NAs and PEG-IFN therapies could be attributed to the distinct mechanisms of action of these treatments. NA treatment primarily works by directly inhibiting viral DNA replication, leading to reduced HBV DNA levels. By contrast, PEG-IFN treatment inhibits viral replication and activates the host immune system to promote viral clearance. Therefore, the reduction in HBV RNA levels during treatment may reflect an enhanced immune response, which is closely associated with a subsequent reduction in HBsAg levels. How the differences in treatment mechanisms and virologic response dynamics affect the relationship between HBV RNA and HBsAg is a subject for future investigation.

The present study has several limitations. Because this was a retrospective study using long-term data collected from existing medical records at a single institution, it may not have captured all relevant variables uniformly. Patient populations and treatment practices vary significantly between institutions and geographical regions. Therefore, the outcomes observed in this study may not be fully representative of those observed in other settings. Future multicenter studies are required to validate our findings and ensure broader applicability. This study is limited by the relatively small number of enrolled patients. Therefore, the conclusions of this study must be confirmed using a larger sample size. In this study, no data were obtained regarding the occurrence of virologic breakthrough or drug resistance. However, we previously reported that serum HBV RNA was a predictive marker for the early emergence of YMDD mutations in patients treated with lamivudine [11]. This suggests that HBV RNA may play a role in predicting drug resistance, although further studies are needed to explore this in patients receiving ETV or other NAs. As we could not measure intrahepatic cccDNA, we could not examine the direct relationship between intrahepatic cccDNA and serum HBV RNA. Although the median observation period in this study was 8.3 years, which is relatively long, it may have been too short to observe HBsAg reduction with NA treatment. Therefore, long-term HBsAg dynamics and eventual treatment efficacy may not have been adequately evaluated.

In conclusion, sustained HBV DNA suppression with long-term NA treatment progressively reduced serum HBV RNA levels in both HBeAg-positive and -negative patients. Serum HBV RNA may serve as a surrogate marker for cccDNA activity in virally suppressed patients receiving ETV treatment. We found that serum HBV RNA levels were valuable for predicting HBeAg seroconversion and favorable virologic responses in HBeAg-positive patients. Conversely, HBcrAg was found to be a better predictor of virologic response than HBV RNA in HBeAg-negative patients. These findings suggest that HBV RNA and HBcrAg have different predictive abilities depending on HBeAg status. Despite similar declines in HBV RNA levels between HBeAg-positive and -negative group, serum HBsAg levels decreased significantly over time in HBeAg-positive group but only after 5 years in HBeAg-negative group. These findings suggest that HBV RNA, HBcrAg, and HBsAg levels may represent distinct aspects of treatment response, and their combination could inform personalized treatment approaches. Further research is needed to investigate the interaction between these markers and other factors to develop precise predictive models for treatment outcomes.

## Supplementary Information

Below is the link to the electronic supplementary material.

Supplementary file1 (DOCX 428 KB)
