# Supplementary material for: Usefulness of serum HBV RNA levels for predicting antiviral response to entecavir treatment in patients with chronic hepatitis B
Source: J Gastroenterol. 2025 Jan 22;60(4):469–78. doi: 10.1007/s00535-025-02211-5 (PMC11922970; doi:10.1007/s00535-025-02211-5)
Supplement: Supplementary file 1 — Supplementary file1 (DOCX 428 KB) [file 535_2025_2211_MOESM1_ESM.docx]

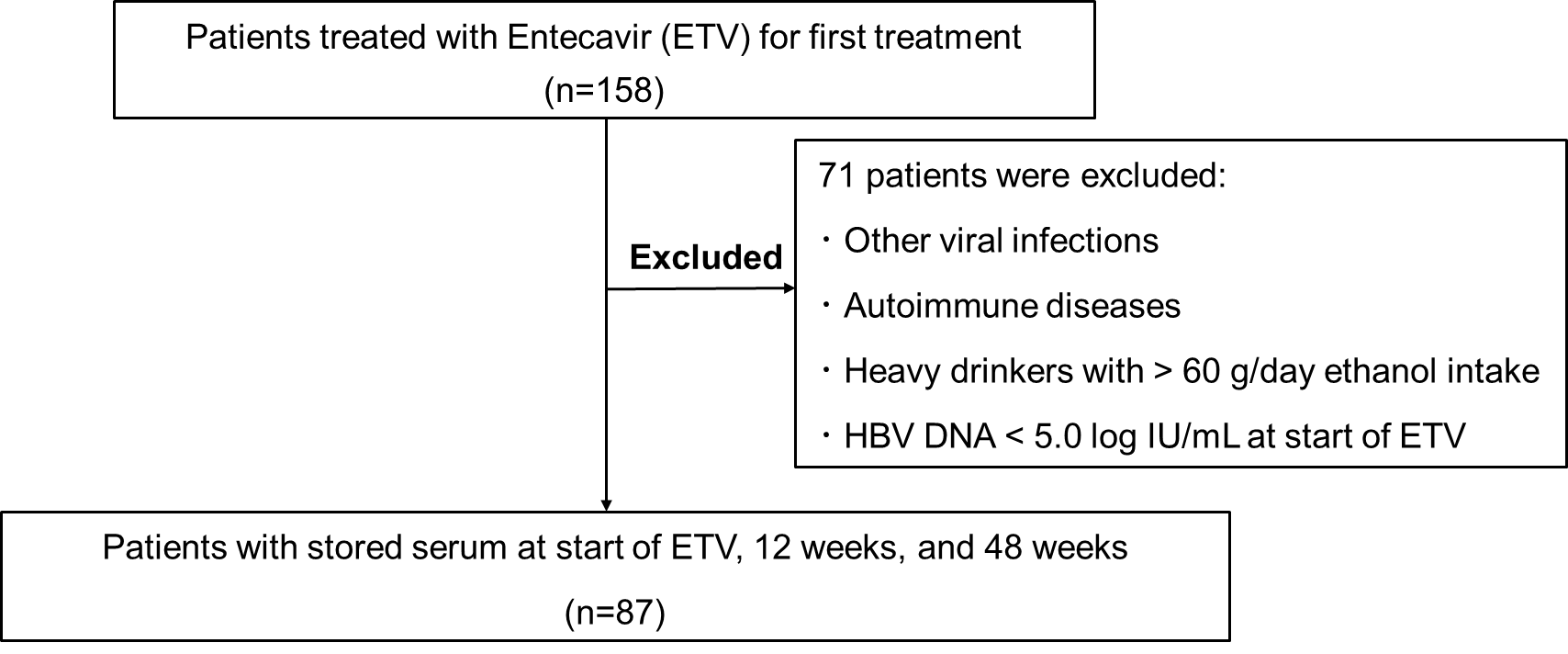


**Supplementary Fig. 1** **Flow chart of participants selection.**


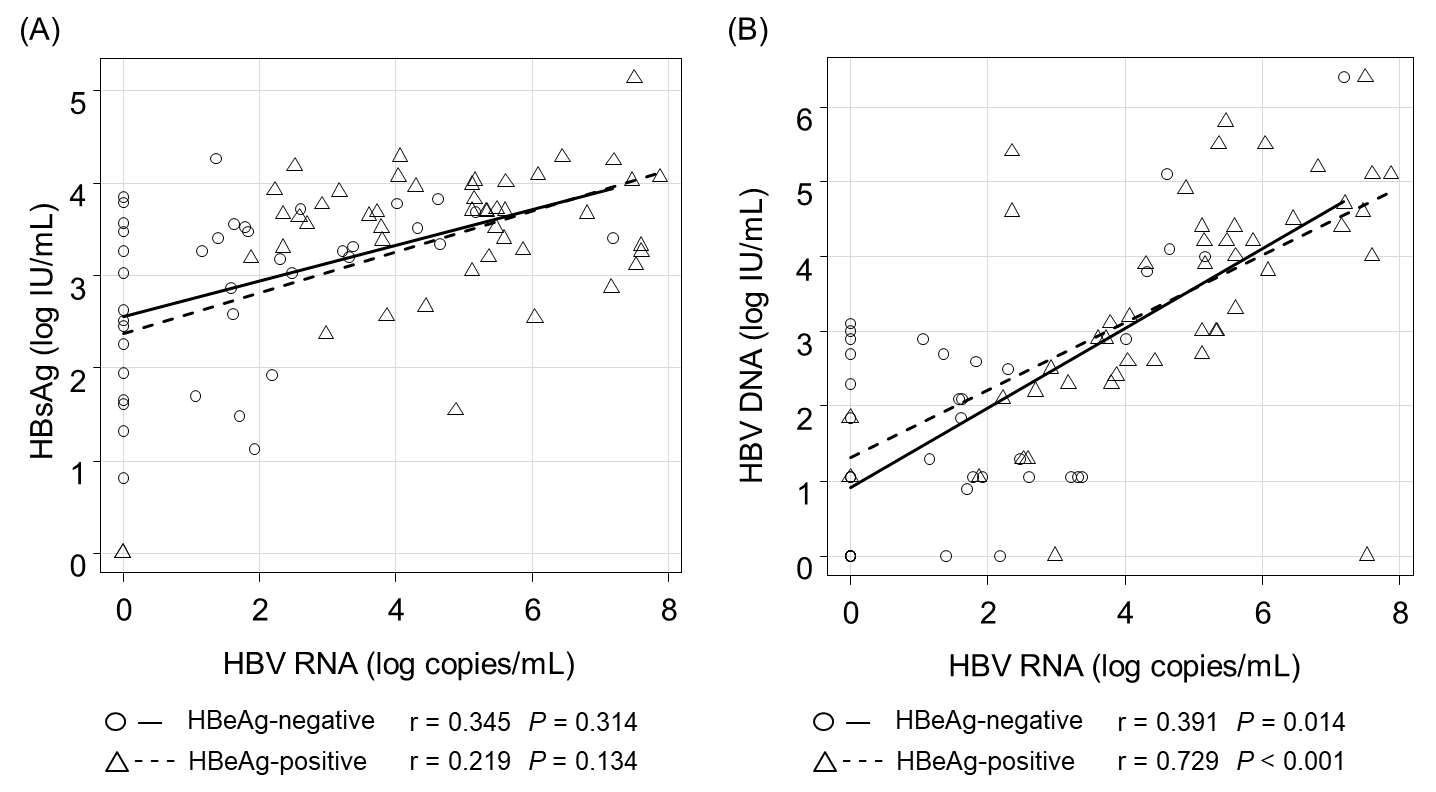


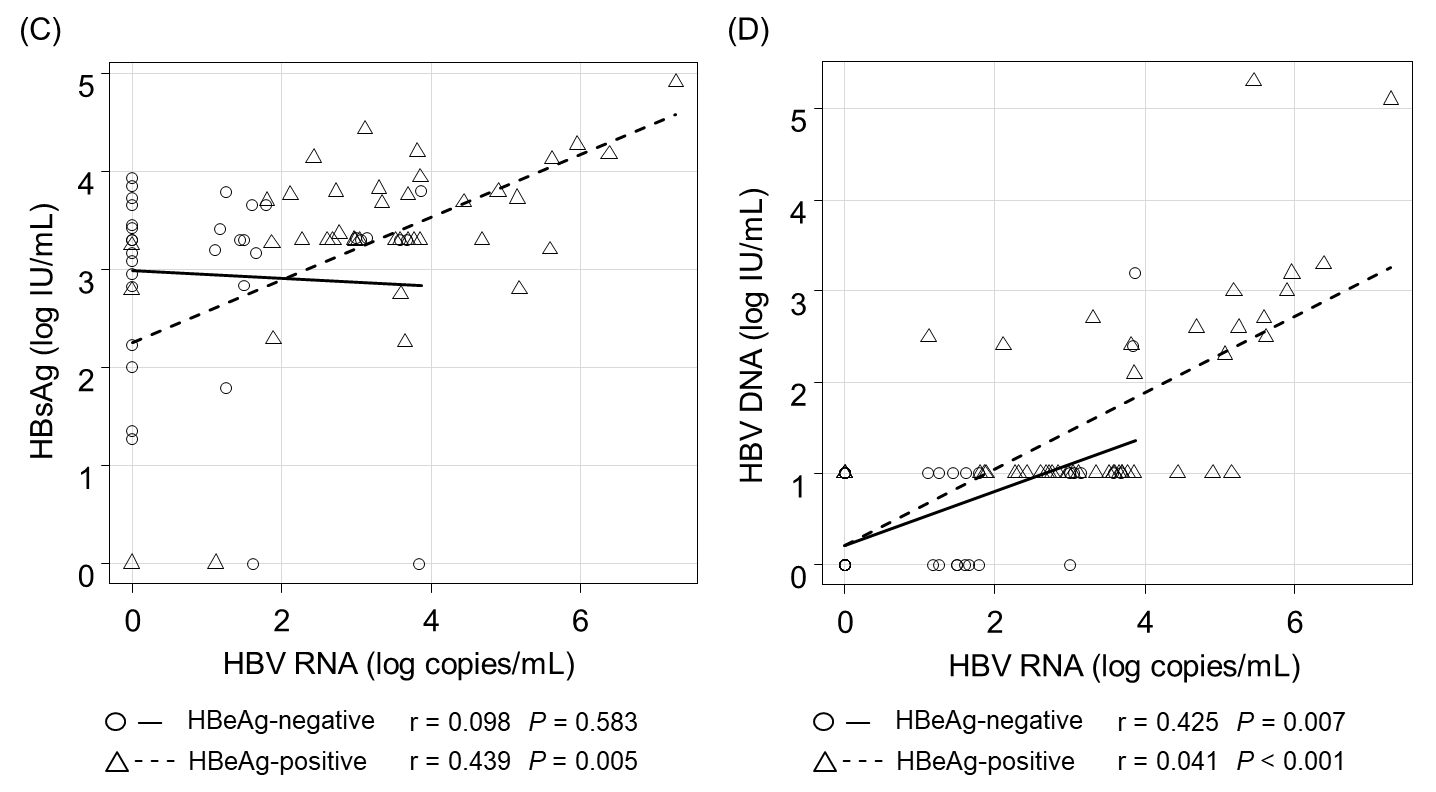


**Supplementary Fig. 2 Correlation between on-treatment hepatitis B virus (HBV) RNA levels and hepatitis B surface antigen (HBsAg) and HBV DNA** **at week 12 and 48 of entecavir (ETV) treatment**

Correlation between HBV RNA and (A) HBsAg at week 12, (B) HBV DNA at week 12, (C) HBsAg at week 48, and (D) HBV DNA at week 48 of ETV treatment.


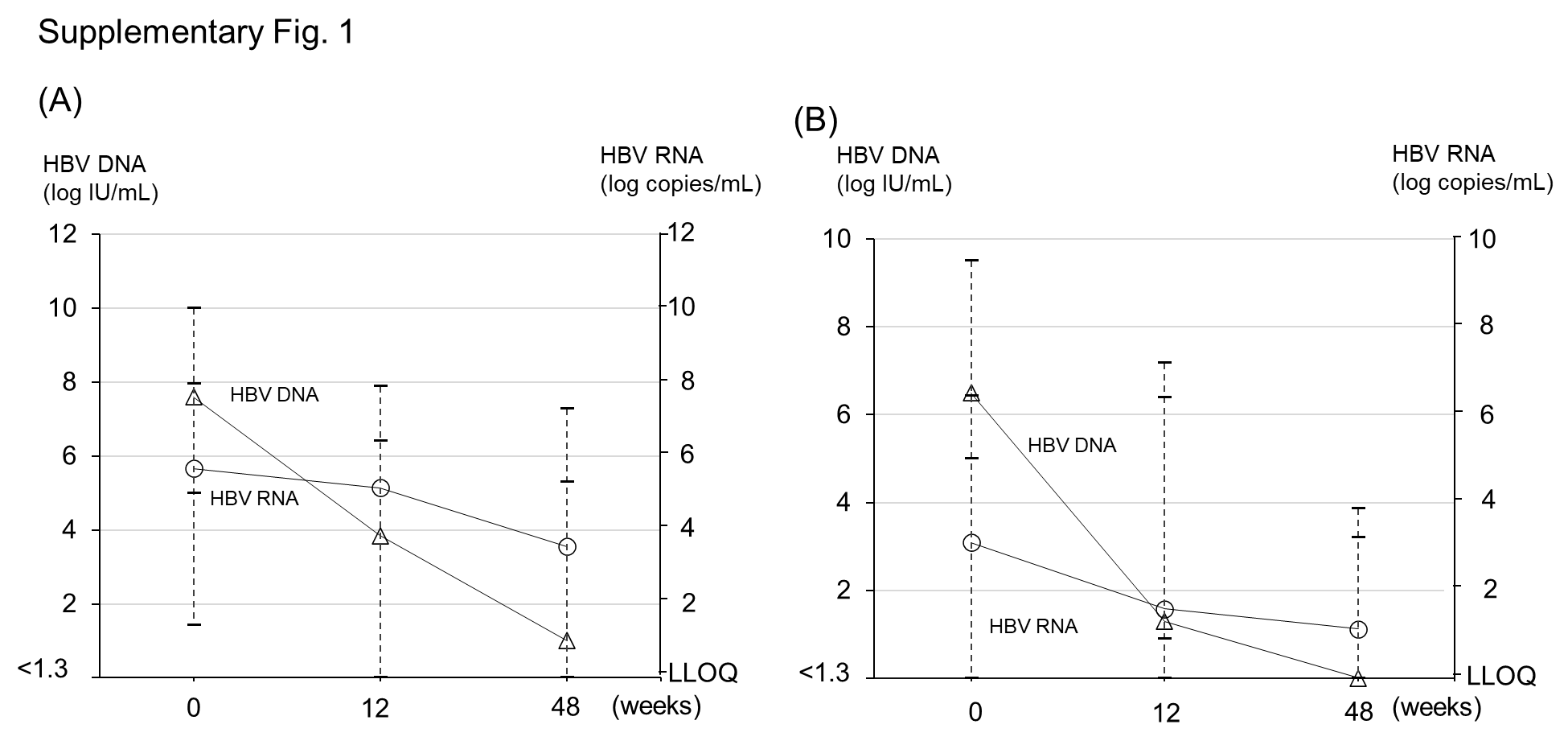


**Supplementary Fig. 3 Changes in hepatitis B virus (HBV) RNA levels and HBV DNA levels during nucleoside/nucleotide analogue (NA) therapy**

(A) hepatitis B e-antigen (HBeAg)-positive and (B) HBeAg-negative group. HBV RNA reduction was gradually compared with HBV DNA reduction.


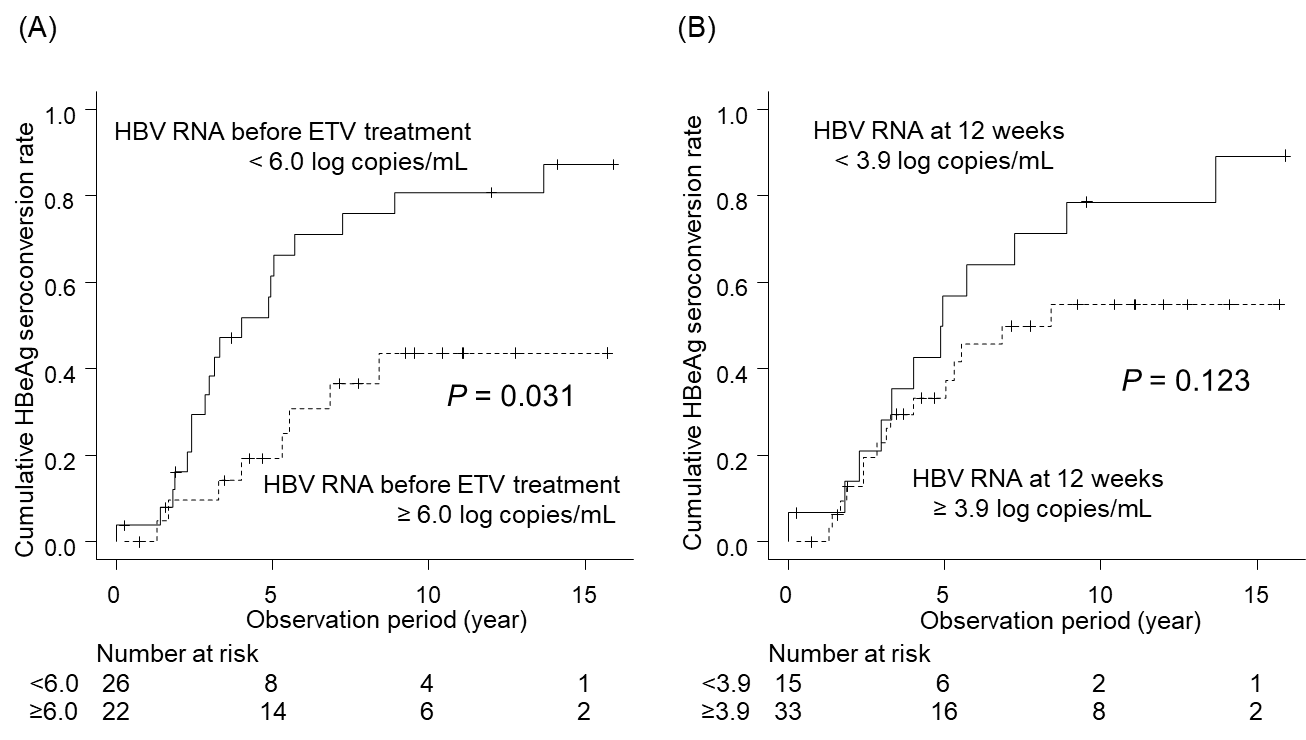


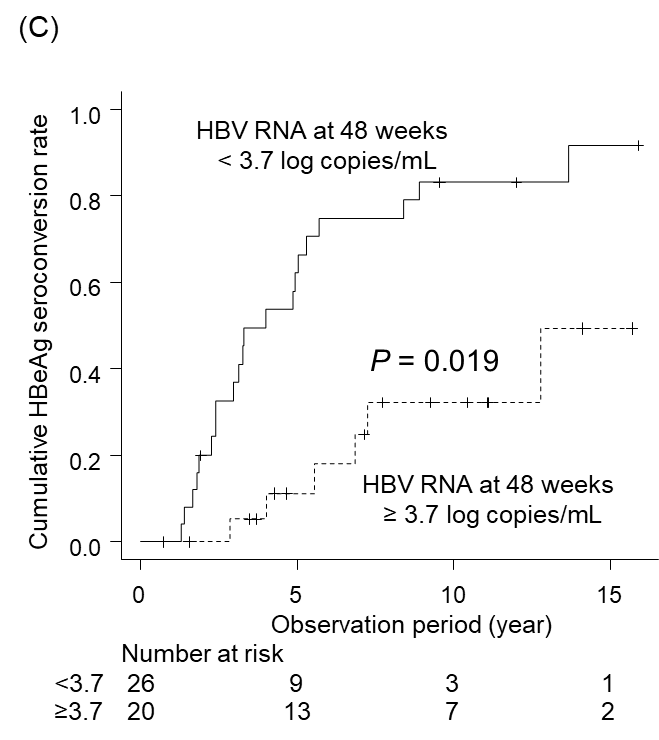


**Supplementary Fig. 4 Cumulative hepatitis B e-antigen (HBeAg) seroconversion rate stratified by baseline and on-treatment HBV RNA levels**

Kaplan-Meier curves showing the cumulative HBeAg seroconversion rate stratified by (A) HBV RNA levels before entecavir (ETV) treatment, (B) HBV RNA levels at 12 weeks of ETV treatment, and (C) HBV RNA levels at 48 weeks of ETV treatment.


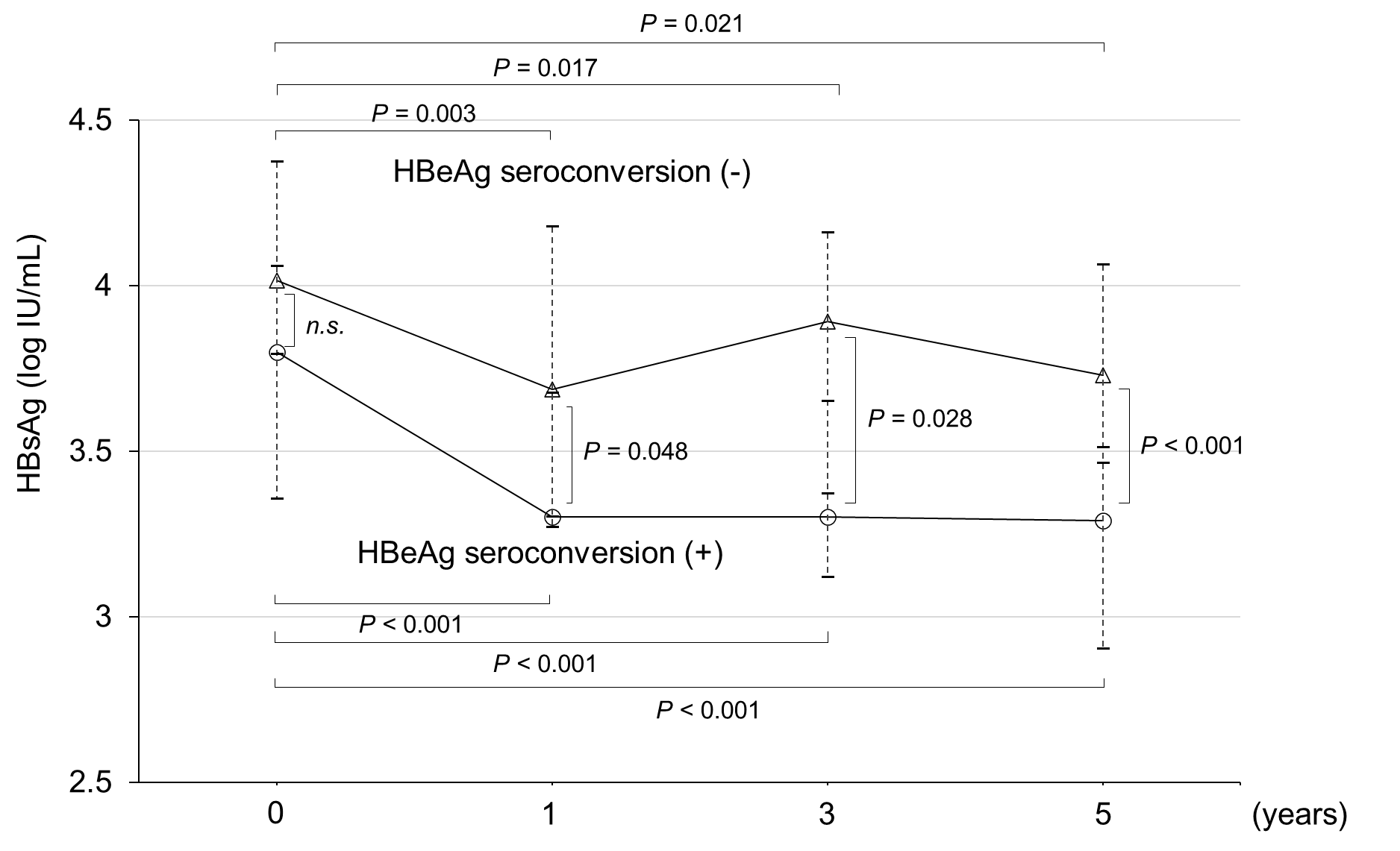


**Supplementary Fig. 5** **Changes in hepatitis B surface antigen (HBsAg) levels with and without hepatitis B e-antigen (HBeAg) seroconversion**

Baseline HBsAg levels were not significantly different between the two groups, but HBsAg decreased significantly in the HBeAg seroconversion group compared to the non-seroconversion group.


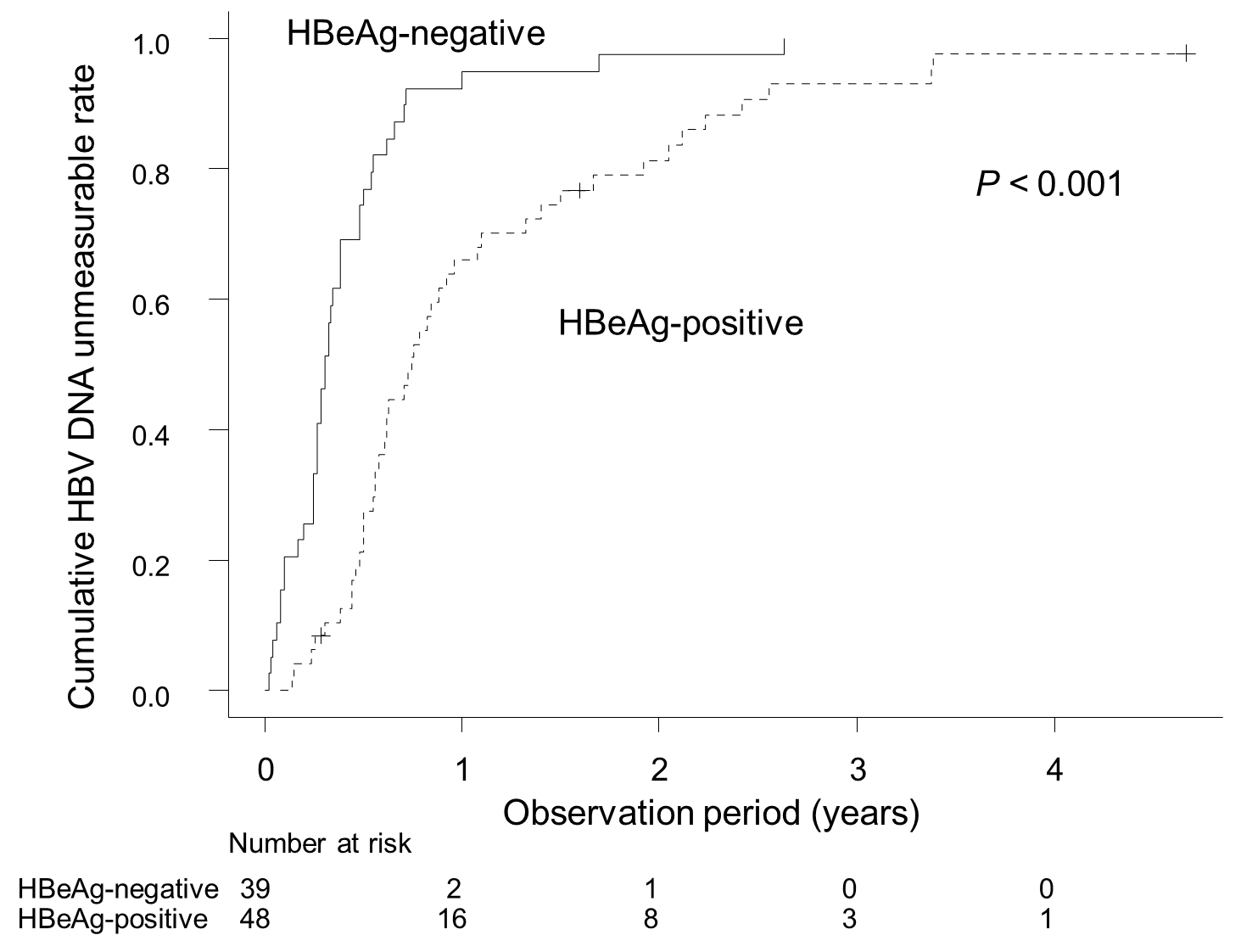


**Supplementary Fig. 6** **Cumulative hepatitis B virus (HBV)-DNA undetectable rate by hepatitis B e-antigen (HBeAg) -positive and -negative**

The HBeAg negative group achieved HBV DNA reduction to undetectable levels earlier than the positive group.


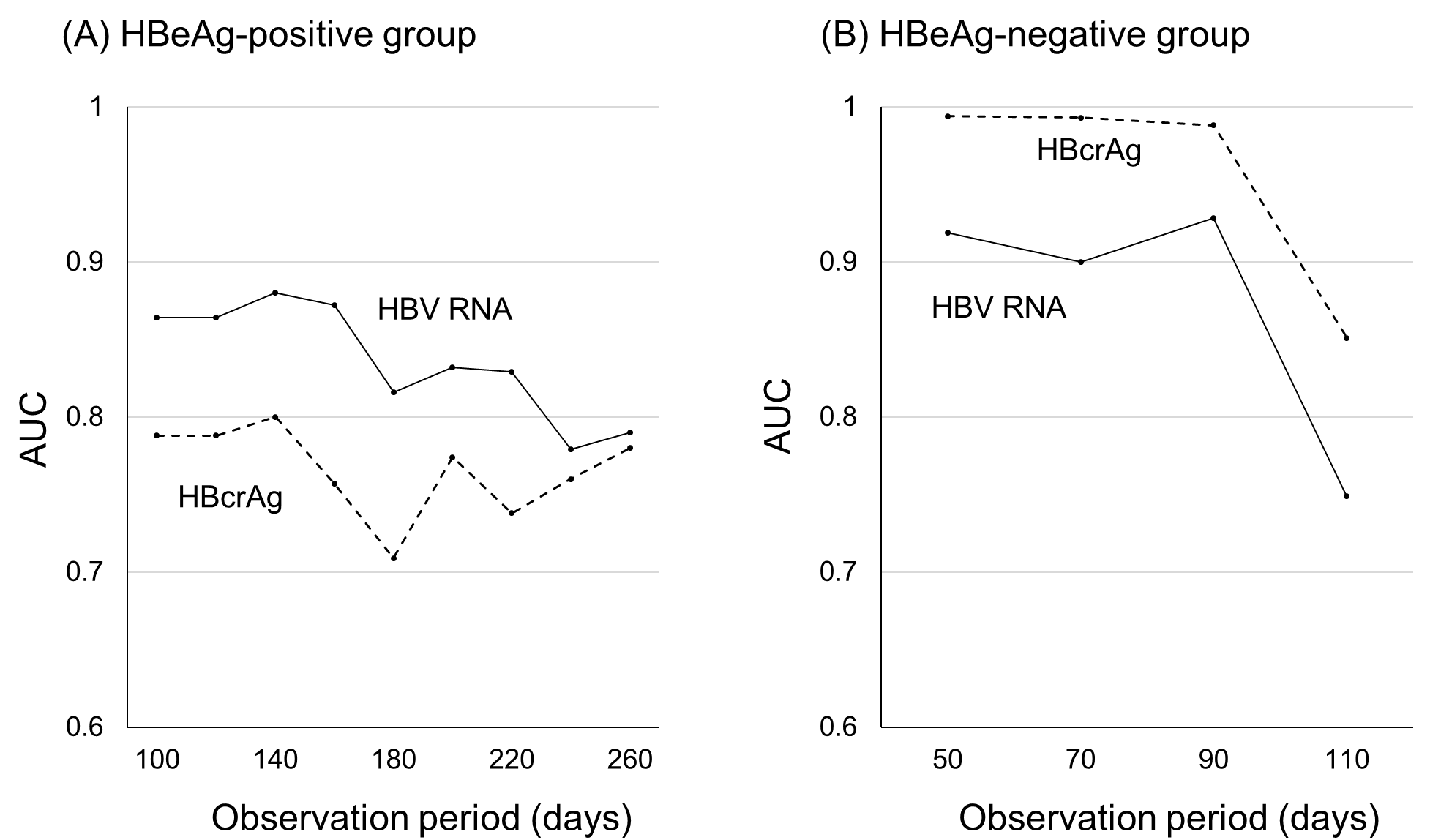


**Supplementary Fig. 7 Time-dependent area under the curve (AUC) of HBV RNA and HBcrAg for predicting virologic response**

(A) HBV RNA had a higher AUC in hepatitis B e-antigen (HBeAg)-positive group. (B) HBcrAg had a higher AUC in HBeAg-negative group.


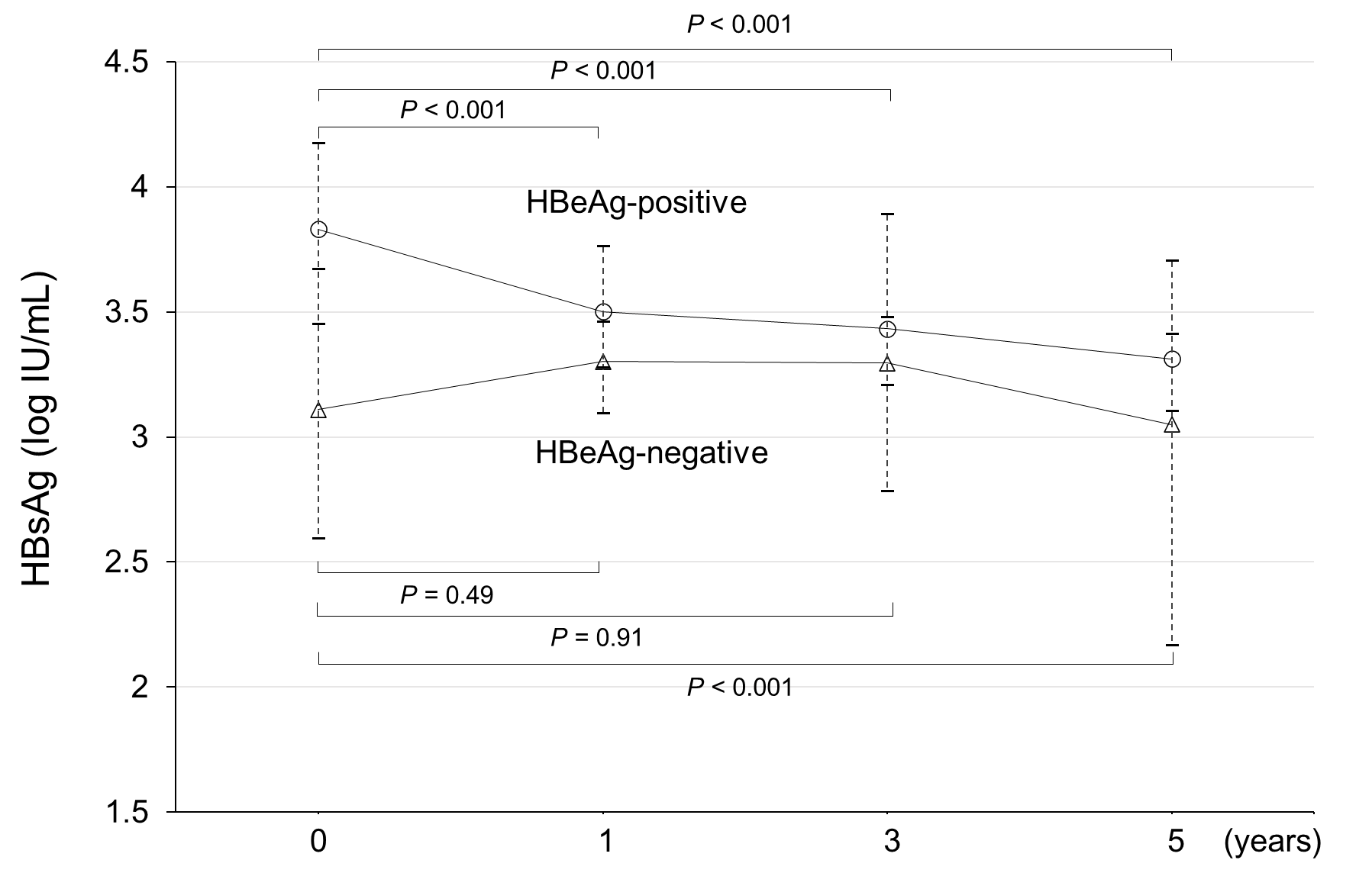


**Supplementary Fig. 8** **Changes in hepatitis B surface antigen (HBsAg) according to** **hepatitis B e-antigen (HBeAg) status**

In the HBeAg-positive group, HBsAg levels decreased significantly from baseline to 1, 3, and 5 years following entecavir (ETV) treatment initiation. In the HBeAg-negative group, HBsAg levels decreased significantly from baseline to 5 years following ETV treatment initiation but were not significantly different from baseline at 1 and 3 years following ETV treatment initiation.
